# Severe Cow’s Milk Allergy Presenting With Life-Threatening Metabolic Acidosis in an Extremely Preterm Infant: A Case Report

**DOI:** 10.7759/cureus.92988

**Published:** 2025-09-23

**Authors:** Nasser Al Shafouri, Mahmoud Khalid, Ahmed Al Muqarshi, Jawaher Al Hatmi

**Affiliations:** 1 Pediatrics and Neonatology, Ibri Hospital, Ibri, OMN

**Keywords:** amino-acid based infant formula, cow’s milk allergy, extremely low birth weight infant, feeding intolerance, neonatal intensive care unit (nicu), preterm neonate, severe metabolic acidosis

## Abstract

Cow's milk allergy (CMA) is a common food allergy in infancy, but its diagnosis in preterm infants remains challenging due to nonspecific symptoms that often mimic complications of prematurity. Early recognition is crucial to prevent significant morbidity in this vulnerable population. We present a case of a male infant born at 27 weeks and four days of gestation with a birth weight of 960 grams. His clinical course was complicated by persistent feeding intolerance, respiratory symptoms, severe metabolic acidosis, and profound growth failure despite multiple interventions for suspected sepsis and necrotizing enterocolitis. On day 45 of life, he developed life-threatening metabolic acidosis (pH: 6.815, bicarbonate: 3.9 mmol/L, base excess: -30.4 mmol/L) with borderline hyperammonemia, initially raising suspicion for an inborn error of metabolism, which was ruled out by normal tandem mass spectrometry.

The infant was initially fed exclusively with expressed breast milk (EBM) from birth. The mother had a normal diet including dairy products throughout the breastfeeding period, providing indirect exposure to cow's milk proteins through breast milk. On day 52 of life, a diagnosis of CMA was considered due to recurrent severe abdominal symptoms and failure to respond to conventional treatments. The infant was switched from EBM to an extensively hydrolyzed, amino acid-based formula (Neocate). Following the dietary change, there was a rapid and dramatic clinical improvement. The metabolic acidosis resolved, feeding intolerance decreased, and the infant demonstrated excellent weight gain (260 grams in eight days). He was successfully discharged home on day 96 of life with a discharge weight of 1.785 kg. This case highlights that severe CMA can present with life-threatening metabolic acidosis in extremely preterm infants, emphasizing the need for high clinical suspicion and early consideration of dietary elimination as both diagnostic and therapeutic intervention. Healthcare providers should consider CMA earlier in the differential diagnosis when standard treatments for common neonatal complications fail to provide adequate clinical improvement.

## Introduction

Cow's milk allergy (CMA) affects 2-3% of infants and is one of the most common food allergies in early childhood, with well-established diagnostic and management guidelines [1-4]. However, diagnosis in preterm infants presents unique challenges and is often significantly delayed [5]. While prevention strategies have been extensively studied [6], the pathophysiology of CMA in preterm infants involves immune system immaturity and increased gut barrier dysfunction, which heighten susceptibility to sensitization from minimal cow's milk protein exposure [7,8]. Høst demonstrated that sensitization can occur in exclusively breastfed infants after exposure to cow's milk formula [9]. The clinical presentation in this vulnerable population frequently overlaps with common complications of prematurity, including necrotizing enterocolitis (NEC) and sepsis, leading to diagnostic confusion [10]. The absence of specific laboratory markers further complicates diagnosis [11]. This diagnostic uncertainty often results in under-recognition of CMA in the preterm population, potentially leading to significant clinical deterioration and growth failure [12].

While prevention strategies have been extensively studied [6], the management of established CMA in extremely preterm infants requires specialized approaches [13]. The overlap between CMA symptoms and common neonatal morbidities, particularly non-IgE-mediated presentations with gastrointestinal symptoms [14], can significantly delay appropriate treatment.

We report a case of severe, life-threatening CMA in a 27-week preterm infant who presented with persistent feeding intolerance and profound metabolic acidosis, ultimately requiring amino acid-based formula for successful management.

## Case presentation

A male infant was delivered at 27 weeks and four days of gestation via emergency caesarean section for maternal preeclampsia with HELLP (Hemolysis, Elevated Liver enzymes and Low Platelets) syndrome. The birth weight was 960 grams, which is appropriate for gestational age. The infant was immediately started on exclusive feeding with expressed breast milk (EBM) from day 1 of life. The mother maintained a normal diet, including regular consumption of dairy products (milk, cheese, yogurt), throughout the breastfeeding period, which provided indirect exposure to cow's milk proteins through her breast milk.

**Figure 1:**
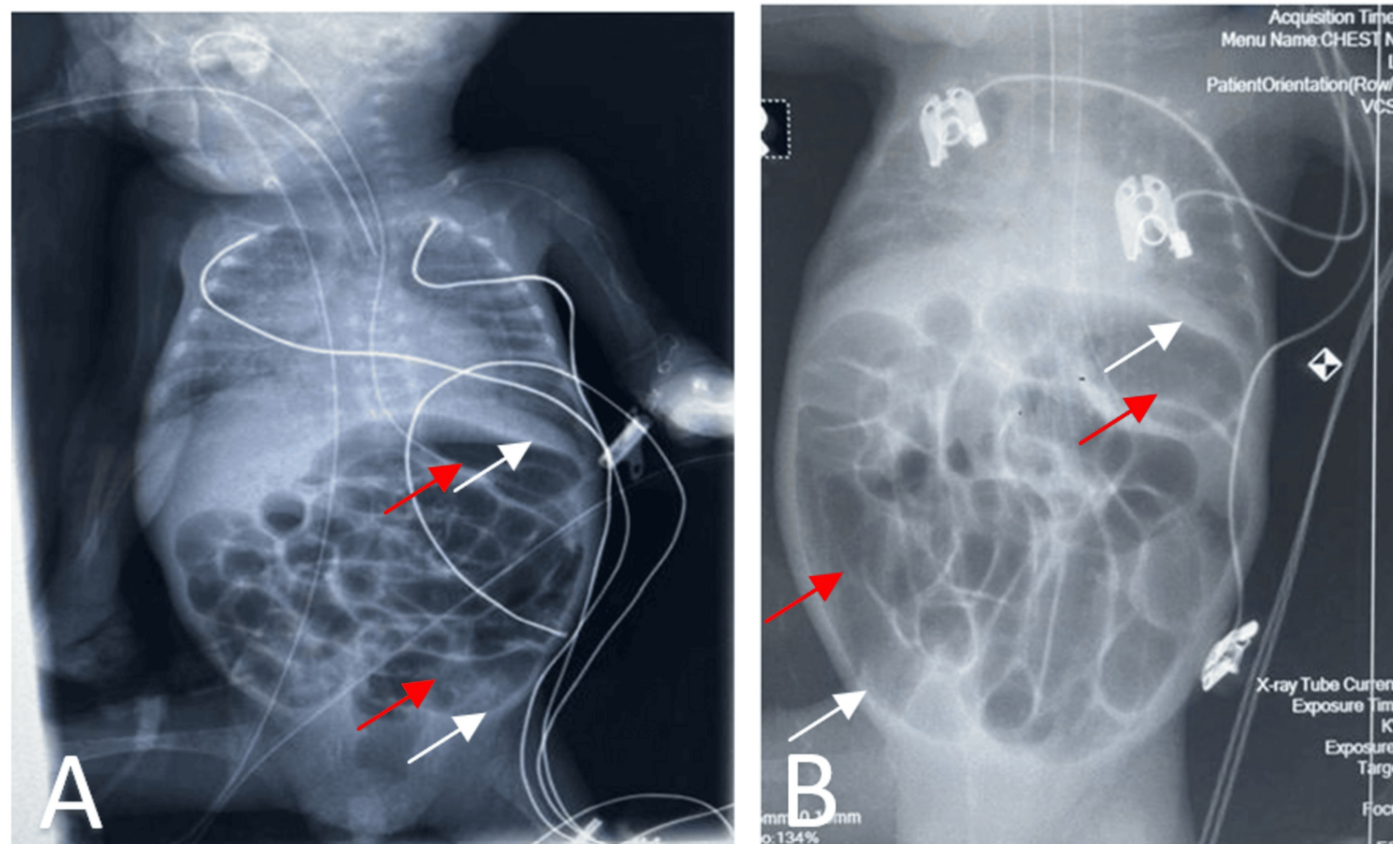
Abdominal radiograph on day 17 (A) and day 41 (B) of life. Radiological images demonstrate persistent bowel distension (indicated by red arrows) without significant evidence of mechanical obstruction or necrotizing enterocolitis (indicated by white arrows), supporting the diagnosis of feeding intolerance secondary to cow's milk allergy.

The infant's clinical course was characterized by significant feeding intolerance with recurrent vomiting and abdominal distension despite exclusive feeding with EBM. Multiple feeding interruptions were required, raising the possibility of NEC. Serial abdominal radiographs showed persistent bowel distension without pneumatosis intestinalis (Figure 1).

He had clinical instability prompting suspicion of sepsis on multiple occasions, but only elevated C-reactive protein (CRP) peaking at 28 mg/L on day 11 of life with multiple negative blood cultures. White blood cell counts fluctuated, but no definitive source of infection was identified despite extensive workup. The metabolic acidosis throughout the neonatal intensive care unit (NICU) stay, with serial blood gas analysis showing consistently abnormal base excess values (ranging from -11 to -6.2 mmol/L) from day 2 through day 38 of life.

On day 45, the infant experienced severe clinical deterioration with profound metabolic acidosis. Initial suspicion of mitochondrial disorder or inborn error of metabolism was raised. However, metabolic screening, including tandem mass spectrometry (TMS) screening, showed normal results, excluding the most common inborn errors of metabolism typically associated with severe metabolic acidosis in neonates.

**Figure 2:**
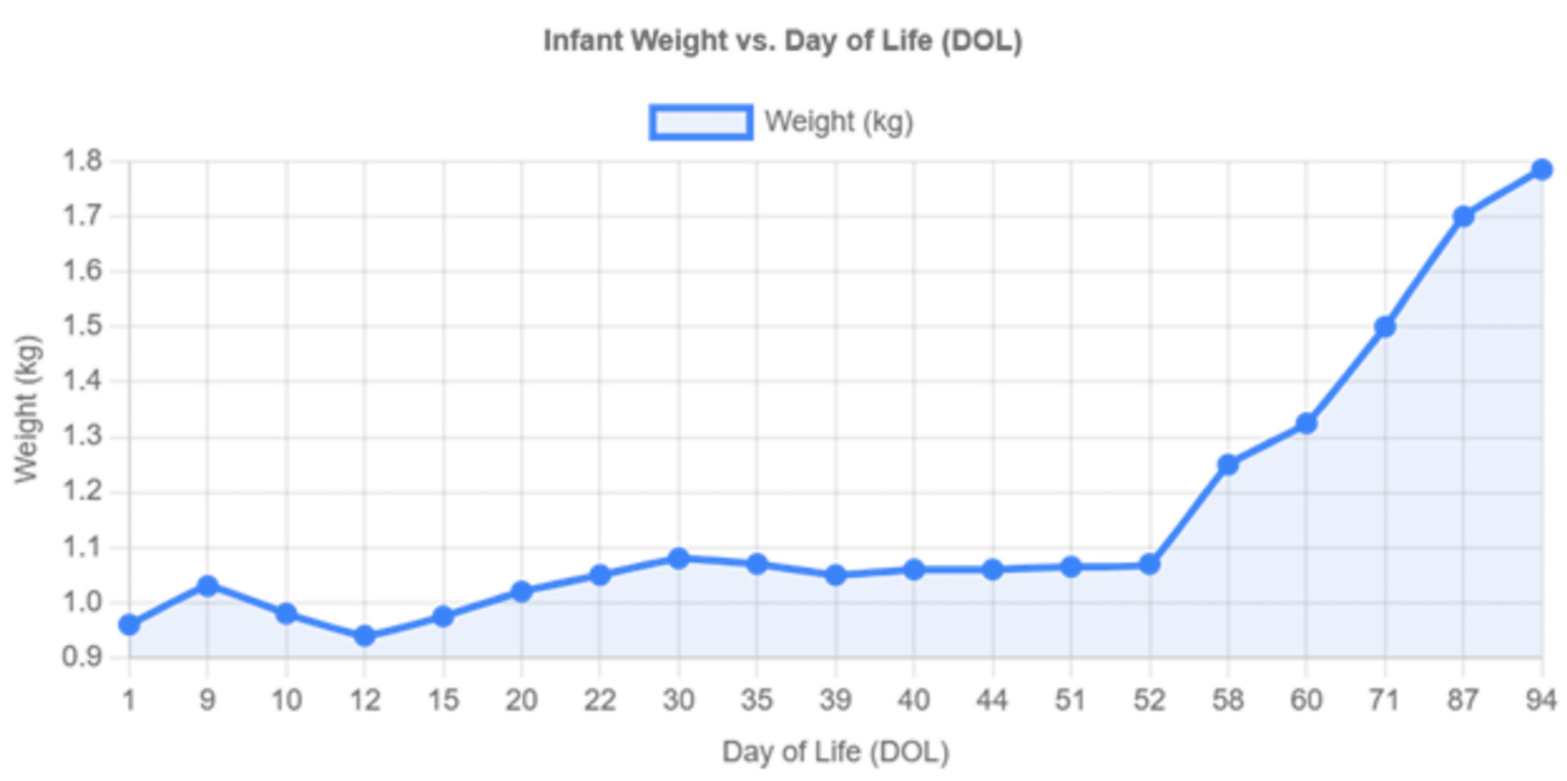
Growth charts showing dramatic improvement. The weight progression chart demonstrates significant growth acceleration following the switch to an amino acid-based formula on day 52, supporting the clinical narrative of CMA diagnosis and successful treatment. CMA: cow's milk allergy

The infant continued to have significant growth failure with net weight gain of only 105 grams over 51 days (Figure 2). Given the persistent, unexplained feeding intolerance and severe metabolic disturbances refractory to standard management, CMA was considered as a differential diagnosis on day 52 of life. The diagnosis was based on the clinical presentation of persistent feeding intolerance, metabolic acidosis, growth failure, and the history of maternal dairy consumption leading to cow's milk protein exposure through breast milk. EBM was discontinued and switched to an amino acid-based formula (Neocate). This decision was made as both a diagnostic and therapeutic intervention.

Following the switch to amino acid-based formula, dramatic clinical improvement occurred with resolution of vomiting and abdominal distension, correction of metabolic acidosis (Table 1), and rapid weight gain of 260 grams in eight days (weight increased from 1.065 kg to 1.325 kg) as demonstrated in Figure 2. The infant continued to thrive on the amino acid-based formula. He was successfully discharged home on day 96 of life with a discharge weight of 1.785 kg, representing appropriate catch-up growth. At discharge, he was clinically stable with resolved feeding intolerance and normal metabolic parameters.

**Table 1:** Laboratory findings.

Parameter	Day 11	Day 45	Day 52	Day 60	Reference Range	Units
pH	7.35	6.815	7.38	7.40	7.35-7.45	-
Bicarbonate	22.1	3.9	21.5	23.2	22-26	mmol/L
Base Excess	-2.1	-30.4	-2.8	-1.5	-2 to +2	mmol/L

## Discussion

This case demonstrates the complex diagnostic challenges posed by severe CMA in extremely preterm infants. The overlap between CMA symptoms and common neonatal morbidities, particularly non-IgE-mediated presentations with gastrointestinal symptoms [14], significantly complicated the diagnostic process. Management of neonatal CMA in high-risk infants requires specialized approaches [13], and appropriate treatment protocols must be followed for optimal outcomes [15].

Our patient's presentation with feeding intolerance and clinical instability, accompanied by an elevated CRP and fluctuating white blood cell counts, led to multiple courses of antibiotics for suspected sepsis, which confounded the clinical picture. The radiographic evidence of significant, persistent bowel distension without signs of mechanical obstruction or NEC further supported a diagnosis of CMA.

The presentation with life-threatening metabolic acidosis (pH: 6.8, base excess: -30 mmol/L) is a rare and unusual manifestation that initially directed the diagnostic workup toward inborn errors of metabolism. However, the lack of supporting biochemical markers and the dramatic clinical resolution following immediate cessation of all enteral feeds, intravenous fluid, empirical antibiotic therapy for suspected sepsis, and the elimination of cow's milk protein from his diet strongly point to CMA as the underlying etiology. The rapid catch-up growth, with a 260-gram weight gain in just eight days after the formula change, serves as compelling evidence. This clear response to an amino acid-based formula is consistent with reports suggesting that severely allergic infants may not tolerate EBM from a mother consuming dairy and require an extensively hydrolyzed formula for recovery [16]. The profound growth failure observed in this case is consistent with previous studies demonstrating the significant impact of CMA on nutritional status and growth parameters in affected infants [12].

The diagnosis was not established until day 52 of life, highlighting significant diagnostic latency that contributed to prolonged morbidity, severe growth failure, life-threatening metabolic complications, and extended hospital stay.

This case supports previous observations that CMA presentation can be delayed in preterm compared to full-term infants [5]. The severity of metabolic acidosis in this case represents an unusual and potentially life-threatening manifestation not commonly reported in the literature.

This case emphasizes the importance of maintaining high clinical suspicion for CMA in preterm infants with unexplained feeding intolerance, with early consideration of elimination diets in refractory cases, understanding that EBM may not be tolerated in severe cases. Furthermore, the recognition of metabolic acidosis can be a presenting feature of severe CMA.

## Conclusions

This case of an extremely preterm infant demonstrates that severe CMA can present atypically with life-threatening metabolic acidosis, a manifestation that can divert clinical suspicion toward metabolic disorders and significantly delay diagnosis. The case underscores the critical importance of maintaining a high index of suspicion for CMA in any preterm infant presenting with unexplained, refractory feeding intolerance, metabolic derangement, and growth failure.

Early consideration and initiation of an elimination diet with an amino acid-based formula can serve as both a diagnostic tool and therapeutic intervention, potentially preventing significant morbidity in this vulnerable population. Healthcare providers caring for preterm infants should consider CMA as a differential diagnosis earlier in the clinical course when standard treatments for common neonatal complications fail to provide adequate clinical improvement.
